# Protective Role of High-Density Lipoprotein Cholesterol in Stress Urinary Incontinence with Special Emphasis on Overweight/Obese Individuals

**DOI:** 10.7150/ijms.116324

**Published:** 2025-07-11

**Authors:** Junlong Huang, Ziqiao Wang, Zheng Liu, Bolong Liu, Wenshuang Li, Xiangfu Zhou

**Affiliations:** Department of Urology, Third Affiliated Hospital of Sun Yat-Sen University, 600 Tianhe Road, Guangzhou 510630, P. R. China.

**Keywords:** Stress urinary incontinence, High-density lipoprotein cholesterol, Obesity, NHANES, Mendelian randomization

## Abstract

**Background:** Increasing evidence shows that lipid metabolism is closely related to the pathogenesis of stress urinary incontinence (SUI). This study aimed to investigate the association between high-density lipoprotein cholesterol (HDL-C) levels and female SUI, evaluate dose-response relationships, and determine the causal effect of HDL-C on SUI risk.

**Materials and methods:** Utilizing cross-sectional data from the National Health and Nutrition Examination Survey (2001-2020, n = 18,415), we assessed the dose-response relationship between HDL-C and SUI using restricted cubic splines and weighted logistic regression. Mendelian randomization (MR) analyses leveraged genetic instruments from European cohorts (HDL-C: n = 9,796; SUI: 5,926 cases/211,672 controls) to infer causality. Subgroup analyses emphasized interactions between HDL-C and BMI.

**Results:** A 1 mg/dL increase in HDL-C was linearly associated with a 0.5% reduction in SUI risk (OR = 0.995, 95% CI: 0.986-0.991, *P* < 0.001). Participants in the highest HDL-C quartile (Q4) exhibited a 25.1% lower SUI risk compared to Q1 (OR = 0.749, 95% CI: 0.652-0.859). Notably, the protective effect of HDL-C was markedly stronger in overweight/obese individuals (BMI ≥ 25 kg/m²: OR = 0.992, *P* = 0.006; BMI ≥ 30 kg/m²: OR = 0.991, *P* = 0.001), with significant interaction (*P* for interaction = 0.015). MR analyses confirmed a causal protective effect of HDL-C on SUI (IVW OR = 0.842, 95% CI: 0.744-0.953), and sensitivity analyses supported robustness.

**Conclusions:** Elevated HDL-C levels are causally linked to reduced SUI risk, with amplified protection in overweight/obese populations. These findings highlight the importance of maintaining healthy HDL-C levels as a targeted strategy for SUI prevention, especially in high-BMI individuals.

## Background

Stress urinary incontinence (SUI), characterized by the involuntary leakage of urine during physical exertion, such as during coughing, sneezing, or laughing 1. It particularly affects women and severely impairs their quality of life 2. Epidemiological studies estimate that 10-40% of women worldwide are affected by SUI 3, 4, with the prevalence increasing with age and obesity 5-7. While well-established risk factors, such as pelvic floor dysfunction 8, estrogen changes 9, and childbirth 7, have been extensively studied, emerging evidence suggests that metabolic health—particularly lipid profiles—may play an underrecognized role in the pathophysiology of SUI 10, 11.

High-density lipoprotein cholesterol (HDL-C) has traditionally been praised for its cardioprotective properties, but recent studies also suggest its involvement in non-cardiovascular conditions, such as sepsis, infections, chronic kidney disease, and diabetes 12. These pleiotropic effects raise the possibility that HDL-C may influence pelvic floor integrity or neuromuscular coordination, potentially reducing the risk of SUI. However, the relationship between HDL-C and SUI remains contentious. An earlier study indicated no association between SUI and cholesterol levels 13. Notably, while a retrospective cohort study by Xu et al. suggested that elevated HDL-C levels might reduce the risk of SUI 4, a Mendelian randomization (MR) analysis by Xiang et al. paradoxically identified a positive association between genetically elevated HDL-C and SUI risk in European women 11. These discrepancies may stem from unaddressed population heterogeneity, particularly the modifying effects of obesity, which is both a key SUI risk factor and a driver of lipid metabolism dysfunction.

To reconcile these inconsistencies, we conducted a dual-evidence study integrating cross-sectional analyses from the National Health and Nutrition Examination Survey (NHANES) and two-sample MR (TSMR). MR leverages genetic variants as instrumental variables (IVs) to infer causal relationships, reducing confounding and reverse causation biases inherent in observational studies 14, 15. By integrating MR with conventional observational analyses, this study strengthens the robustness of causal inference. Our objectives were threefold: (1) To evaluate the association between HDL-C levels and SUI prevalence, with specific emphasis on overweight/obese individuals (BMI ≥ 25 kg/m²); (2) To explore whether BMI modifies the protective effect of HDL-C through interaction analyses, and (3) To infer causality using genetic instruments, while rigorously controlling for potential pleiotropy and reverse causation.

## Materials and Methods

### Study population in NHANES

**Figure 1:**
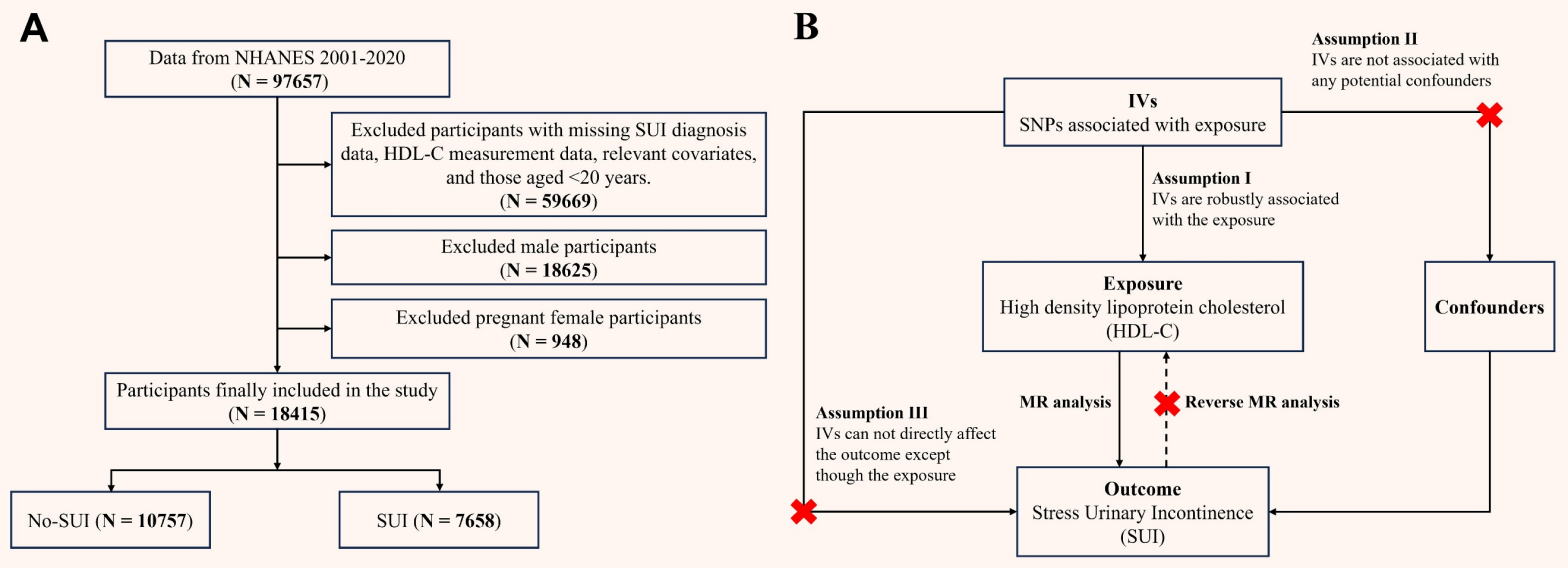
** (A) Flowchart of participant enrollment from NHANES 2001-2020; (B) Schematic of Mendelian randomization.** NHANES: national health and nutrition examination survey; HDL-C: high density lipoprotein cholesterol; SUI: stress urinary incontinence; MR: mendelian randomization; SNPs: single nucleotide polymorphisms; IVs: instrumental variables.

NHANES is a biennial nationwide survey assessing health and nutritional status of non-institutionalized U.S. residents. Participants underwent interviews and physical examinations at Mobile Examination Centers (MECs), with blood samples analyzed at certified laboratories (University of Minnesota). NHANES data are publicly available on the CDC website (https://www.cdc.gov/nchs/nhanes/), and the survey protocol was approved by the Institutional Review Board of the National Center for Health Statistics, with informed consent obtained from all participants. To focus on obesity-related metabolic effects, we included women aged ≥ 20 years from cycles 2001-2020, excluding males, pregnant women, and individuals missing SUI or HDL-C data. The 2019-2020 cycle was excluded due to COVID-19 disruptions, replaced by the combined 2017-March 2020 pre-pandemic data. BMI stratification followed WHO criteria 16: normal weight (< 25 kg/m²), overweight (25-30 kg/m²), and obese (≥ 30 kg/m²). Final analysis included 18,415 participants (Figure 1A).

### Data sources and study population in TSMR

We used NHGRI-EBI Catalog (https://gwas.mrcieu.ac.uk/) to download the genome-wide association study (GWAS) datasets. The genome-wide dataset for HDL-C (GCST005058) was derived from the UK Household Longitudinal Study, with 9,796 European ancestry participants 17. The SUI dataset (GCST90436516) was sourced from the UK Biobank, which recruited British white female participants aged 40-69 years. Cases of SUI were identified using a comprehensive framework that included the ICD-10 (N39.3), as well as self-reported operation codes (total: 5,926 cases and 211,672 controls) 18, 19.

### SUI assessment in NHANES

SUI was defined based on participants' self-reported responses to the question: "During the past 12 months, have you leaked or lost control of even a small amount of urine during activities such as coughing, lifting, or exercising?" The frequency of SUI was determined by the answer to the question 5: "How frequently does this occur?" Responses of "less than once a month" and "a few times a month" are classified as frequency 1, "a few times a week" as frequency 2, and "every day and/or night" as frequency 3.

### HDL-C measurement in NHANES

HDL-C levels were directly measured in serum samples. Detailed instructions on sample collection and processing can be found in the NHANES Laboratory Procedures Manual. The contracted laboratory adhered to Westgard rules for quality control. NHANES quality assurance and quality control procedures comply with the 1988 Clinical Laboratory Improvement Amendments standards, ensuring reliable data.

### Other covariates used in NHANES

To control for potential confounding effects, the following demographic characteristics were adjusted: age, race (non-Hispanic white, Mexican American, other Hispanic, non-Hispanic black, other race), Education (Less than high school, High school, Greater than high school), marital status (married/living with partner, living alone), poverty-income ratio (PIR, < 2, ≥ 2), hypertension (yes, no), diabetes (yes, no), vigorous recreational activities (yes, no), smoke (never, former, current), alcohol use (yes, no), coronary heart disease (CHD, yes, no), stroke (yes, no), cancer/malignancy (yes, no), and other relevant metabolic biomarkers, such as total cholesterol (TC), alanine aminotransferase (ALT), aspartate aminotransferase (AST), albumin (ALB), serum creatinine (SCr), etc. Covariates were pre-selected based on known or suspected confounders between HDL-C and SUI 5, 20-23. BMI was included both as a confounder and an effect modifier, with explicit stratification in subgroup analyses. Detailed data collection procedures are available on the NHANES website (https://wwwn.cdc.gov/nchs/nhanes).

### Screening of genetic instrumental variables in TSMR

MR relies on three core assumptions to assess causality between an exposure and outcome: (1) the genetic variant must be strongly associated with the risk factor; (2) genetic variant must not be associated with any known or unknown confounders; (3) genetic variant must influence the outcome solely through the risk factor, not via other pathways 24. To satisfy these, we selected IVs as follows: First, single nucleotide polymorphisms (SNPs) associated with HDL-C (exposure) at genome-wide significance (*P* < 5 × 10⁻⁸) were identified; For reverse MR (SUI as exposure), a lenient threshold (*P* < 1 × 10⁻⁶) ensured sufficient IVs. Second, we excluded SNPs in linkage disequilibrium (r² < 0.001, kb = 10,000) using European ancestry reference data. Third, instrument strength was validated via F-statistics (F > 10 for all SNPs, minimizing weak instrument bias) 25. Fourth, palindromic SNPs with intermediate allele frequencies were excluded, and the exposure and outcome datasets were harmonized using effect allele frequencies. Fifth, to address potential confounding, we used the NHGRI-EBI Catalog (https://www.ebi.ac.uk/gwas/) to identify and eliminate SNPs associated with confounding factors. Finally, Steiger filtering was applied to eliminate SNPs with reverse causality, ensuring directional plausibility.

### Statistical analysis

All analyses accounted for the complex sampling design of NHANES using appropriate sampling weights. Continuous variables were expressed as weighted means ± standard deviations, and categorical variables as weighted frequencies (percentages). Group differences were assessed via weighted t-tests/ANOVA for continuous variables and weighted chi-square tests for categorical variables.

To evaluate the dose-response relationship between HDL-C and SUI, we employed restricted cubic splines (RCS) with 3 knots, using the median HDL-C level as the reference. To quantify the strength of the association between HDL-C and SUI, we constructed a stepwise weighted multivariable logistic regression model: Model 1 (unadjusted), Model 2 (adjusted for age, race, marital status, education, and poverty-income ratio), and Model 3 (additionally adjusted for BMI, hypertension, diabetes, lifestyle factors, comorbidities, and metabolic biomarkers including TC, ALT, AST, ALB, SCr, BUN and TBil). HDL-C was analyzed as a continuous variable, a dichotomized variable (median cutoff: 67 mg/dL), and categorized into four groups defined by quartile boundaries (Q1: < 25^th^; Q2: 25^th^ - < 50^th^; Q3: 50^th^ - < 75^th^; Q4: ≥ 75^th^ percentile).

Additionally, subgroup and interaction analyses were conducted to assess the effect of HDL-C across different subgroups, such as age, race, education, BMI, hypertension, diabetes, smoke, alcohol use, and other health conditions. Sensitivity analyses were performed to assess the robustness of the results, considering potential influences from data release cycle, blood sampling time, and LDL-C. We also performed further sensitivity analysis on the leakage frequency of SUI to explore the association between HDL-C and different frequencies of SUI.

In the TSMR analysis, the primary analysis used inverse variance weighting (IVW) 26, supplemented with weighted median, weighted mode, MR-Egger, and simple mode methods. Although IVW provides precise estimates, it may be susceptible to bias due to IVs or pleiotropy 27. To ensure the robustness of our results, we performed several sensitivity analyses. First, MR-Egger regression and the MR-Pleiotropy RESidual Sum and Outlier (MR-PRESSO) method were used to assess potential horizontal pleiotropy, with a *P*-value > 0.05 suggesting no significant pleiotropy 28, 29. Second, Cochrane's Q test was employed to evaluate heterogeneity among the IVs, *P* > 0.05 indicating no significant heterogeneity 26. Finally, the leave-one-out analysis was performed to evaluate the influence of individual SNPs on MR analysis.

In this study, two-sided *P* < 0.05 considered statistically significant. All analyses were performed using R software (version 4.4.1, http://www.R-project.org), with the “TwoSampleMR” and “MR-PRESSO” packages used for two-sample MR analysis.

## Results

### Participant characteristics​

A total of 18,415 women from NHANES (weighted population: 85,864,847, Table S1 in Supplementary materials) were included, of whom 7,658 (41.59%) reported SUI (Table 1). Compared to the non-SUI group, women with SUI were older (41.12% aged 40-59 years; 36.85% aged ≥ 60 years), had a higher prevalence of obesity (47.35% vs. 36.00%), hypertension (42.03% vs. 31.42%), and diabetes (14.99% vs. 9.80%), and exhibited elevated metabolic markers (all *P* < 0.001). Notably, HDL-C levels were significantly lower in the SUI group (56.31 ± 16.17 mg/dL vs. 58.77 ± 16.51 mg/dL; *P* < 0.001), with a dose-dependent reduction in SUI risk across HDL-C quartiles (*P* < 0.001).

### HDL-C and SUI associations​

**Figure 2:**
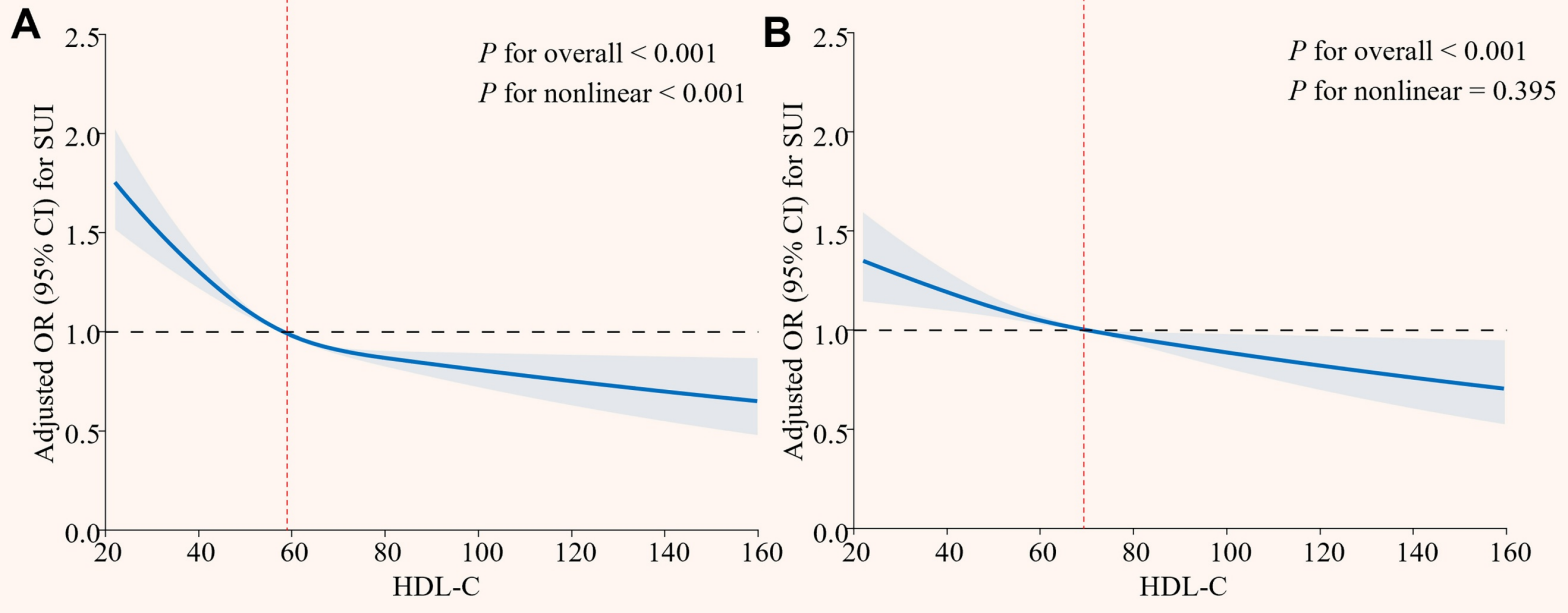
** Restricted cubic spline analysis of the association between HDL-C and SUI. (A) Model 1; (B) Model 3.** HDL-C: high density lipoprotein cholesterol; SUI: stress urinary incontinence; OR: odds ratio; CI: confidence intervals. Model 1 was an unadjusted crude model. Model 3 adjusted for age, race, marital status, PIR, education, BMI, hypertension, diabetes, alcohol use, smoke, vigorous recreational activities, coronary heart disease, stroke, cancer/malignancy, TC, ALT, AST, ALB, SCr, BUN, and TBil.

RCS analysis revealed a nonlinear association between HDL-C and SUI in the crude model (*P* for overall < 0.001, *P* for nonlinear < 0.001), with a steep risk decrease at lower HDL-C levels and a plateau in risk reduction at higher HDL-C levels (Figure 2A). After full adjustment, the relationship became linear (*P* for overall < 0.001, *P* for nonlinear = 0.395; Figure 2B).

Stepwise weighted multivariate logistic regression showed that, for each 1 mg/dL increase in HDL-C, the risk of SUI decreased by 0.8% (95% CI: 0.989 - 0.994, *P* < 0.001) in the unadjusted model (Table 2). This remained significant in both the partially adjusted (OR = 0.988, 95% CI: 0.986-0.991, *P* < 0.001) and fully adjusted models (OR = 0.995, 95% CI: 0.992-0.998, *P* < 0.001). Participants with HDL-C ≥ 67 mg/dL had a 17.3% (95% CI: 0.747 - 0.917) lower risk of SUI. Furthermore, a stepwise reduction in the risk of SUI with increasing HDL-C levels (Q1 → Q4) (*P* for trend < 0.001). In the fully adjusted model, the risk of SUI in the highest HDL-C level (Q4) was 25.1% (95% CI: 0.652-0.859) lower compared to the lowest level (Q1), while the second level (Q2) showed no significant protective effect (OR = 0.950, 95% CI: 0.847 - 1.066, *P* = 0.380). Additionally, no significant associations were observed for total cholesterol or LDL-C (Table S2 in Supplementary materials).

### Subgroup analyses

**Figure 3:**
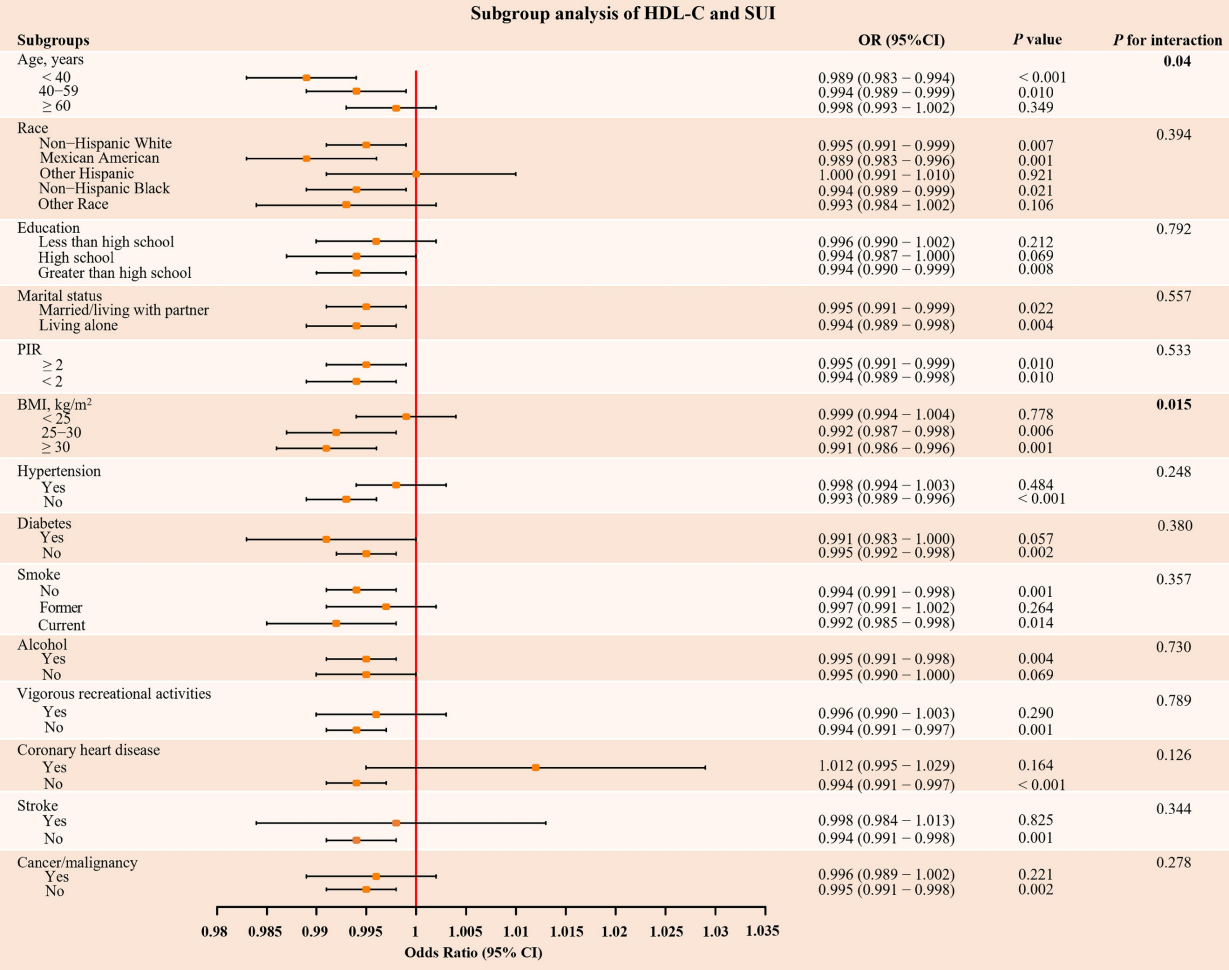
** Subgroup analysis of the association between HDL-C and SUI.** HDL-C: high density lipoprotein cholesterol; SUI: stress urinary incontinence; PIR: poverty-income ratio; BMI: body mass index; OR: odds ratio; CI: confidence intervals. The orange dots and their corresponding black solid lines represent the specific OR values and their 95% CI associated with the risk of SUI in each subgroup, the red vertical line represents the reference line for OR = 1. All models were adjusted for age, race, marital status, PIR, education, BMI, hypertension, diabetes, alcohol use, smoke, vigorous recreational activities, coronary heart disease, stroke, cancer/malignancy, TC, ALT, AST, ALB, SCr, BUN, and TBil. *P*-value < 0.05 was considered significant.

The results of subgroup analysis revealed that consistent inverse associations between HDL-C and SUI across demographic and clinical strata (Figure 3). Critically, the protective effect of HDL-C was significantly stronger in overweight/obese individuals (BMI ≥ 25 kg/m²: OR = 0.992, *P* = 0.006; BMI ≥ 30 kg/m²: OR = 0.991, *P* = 0.001), with a significant interaction effect (*P* for interaction = 0.015). Similarly, younger women (< 60 years) exhibited a more pronounced risk reduction (*P* for interaction < 0.05). Additional subgroup analyses that converted HDL-C to dichotomous and quaternary variables also showed consistent results. (Table S3 and S4 in Supplementary materials). Sensitivity analyses adjusting for the data release cycle, blood sampling time, and LDL-C levels further supported the robustness of these findings (Table S5, S6, and S7 in Supplementary materials).

**Figure 4:**
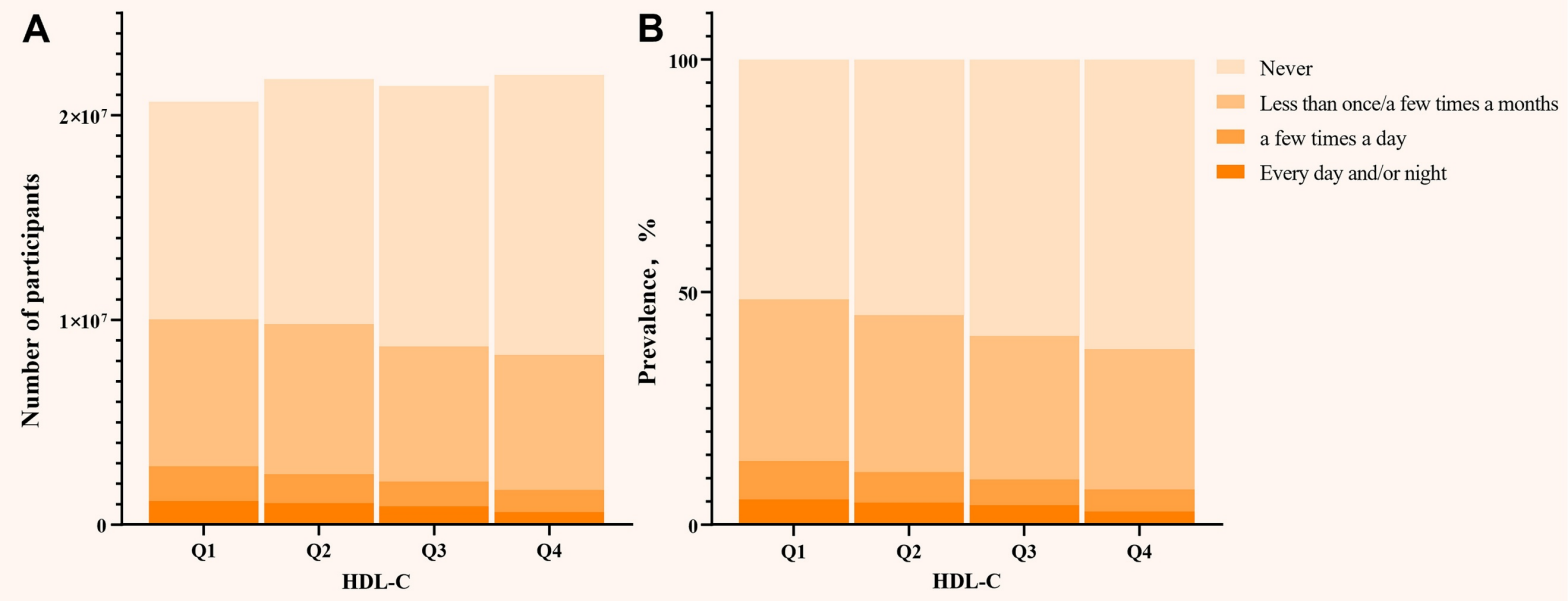
** (A) Distribution of the number of participants in different HDL-C quartiles; (B) The prevalence of SUI in different HDL-C quartiles.** HDL-C: high density lipoprotein cholesterol; SUI: stress urinary incontinence. All models were adjusted for age, race, marital status, PIR, education, BMI, hypertension, diabetes, alcohol use, smoke, vigorous recreational activities, coronary heart disease, stroke, cancer/malignancy, TC, ALT, AST, ALB, SCr, BUN, and TBil.

Notably, when SUI was stratified by different leakage frequencies, HDL-C levels were inversely associated with all frequency levels of SUI (Figure 4). Increasing HDL-C levels (Q1 → Q4) led to a parallel decrease in the frequency of SUI. Specifically, for each 1 mg/dL increase in HDL-C, the risk of frequency 1 decreased by 0.6% (95% CI: 0.991 - 0.998, *P* = 0.001), frequency 2 by 1.0% (95% CI: 0.985 - 0.995, *P* < 0.001), and frequency 3 by 0.9% (95% CI: 0.984 - 0.999, *P* = 0.023). Participants with HDL-C ≥ 67 mg/dL had a 16.9% (95% CI: 0.741 - 0.931, *P* = 0.002) lower risk of frequency 1, 28.7% (95% CI: 0.582 - 0.873, *P* = 0.001) lower risk of frequency 2, and 37.4% (95% CI: 0.497 - 0.787, *P* < 0.001) lower risk of frequency 3 compared to those with HDL-C < 67 mg/dL. Furthermore, in the highest HDL-C level (Q4), the risk of mild, moderate, and frequency 3 decreased by 25.0% (95% CI: 0.647 - 0.870, *P* < 0.001), 40.2% (95% CI: 0.472 - 0.756, *P* < 0.001), and 41.2% (95% CI: 0.446 - 0.775, *P* < 0.001), respectively (Table S8 in Supplementary materials).

### Mendelian randomization analysis​

**Figure 5:**
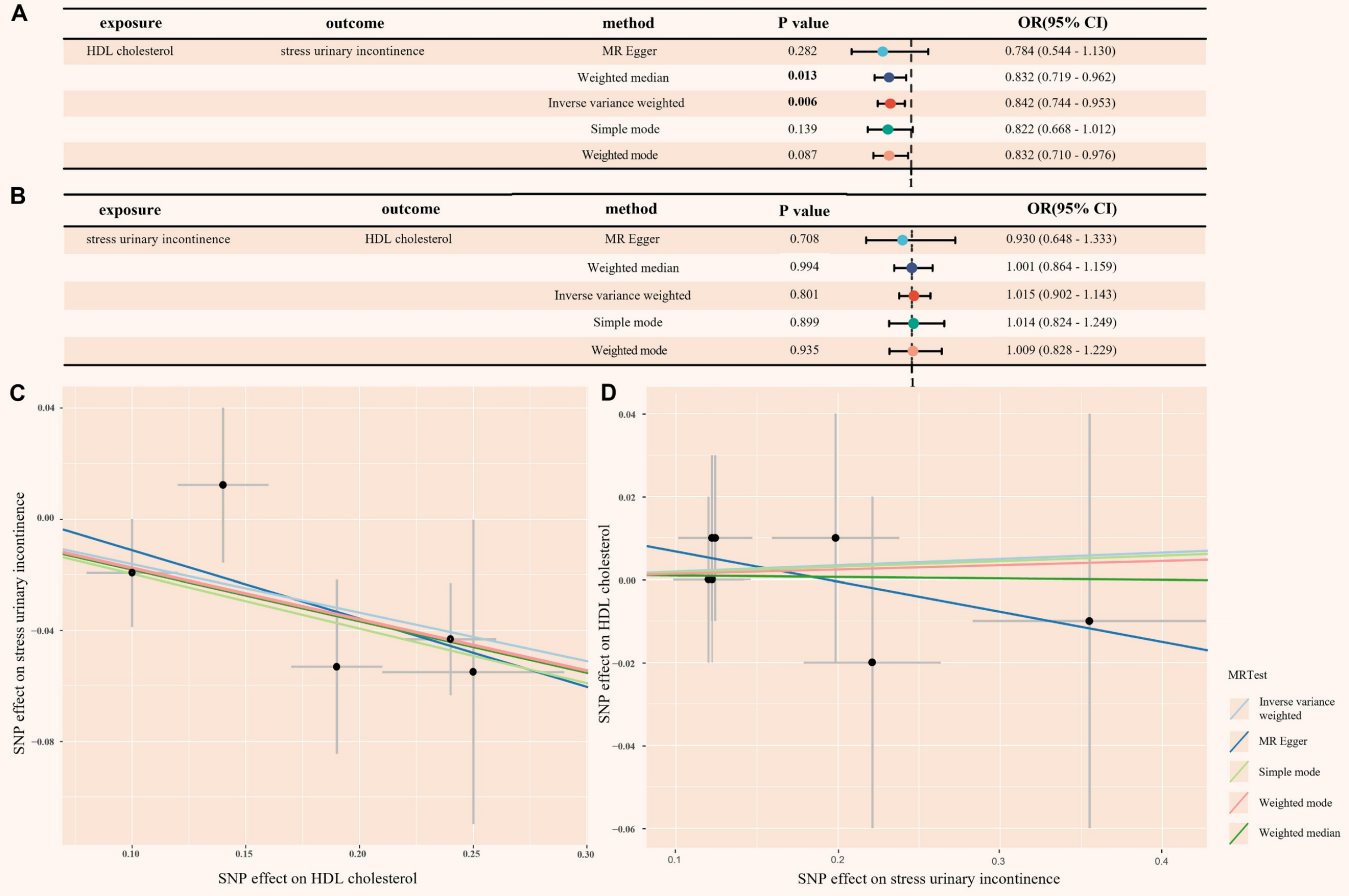
** (A) Mendelian randomization causal effect analysis of HDL-C and SUI; (B) Mendelian randomization for reverse causal effect between HDL-C and SUI; (C) Scatter plot of the bidirectional association between SNP effect and causal relationship between HDL-C and SUI; (D) Scatter plot of the bidirectional association between SNP effect and the reverse causal relationship between HDL-C and SUI.** HDL cholesterol: high density lipoprotein cholesterol; SUI: stress urinary incontinence; MR: mendelian randomization; SNP: single nucleotide polymorphism; OR: odds ratio; CI: confidence intervals.

When HDL-C was used as the exposure instrument, five IVs were identified (Table S9 in Supplementary materials). In the IVW analysis, a significant inverse association was observed between HDL-C and SUI risk (OR = 0.842, 95% CI = 0.744 - 0.953; *P* = 0.006), as shown in Figure 5. Additionally, no evidence of heterogeneity or horizontal pleiotropy was found, as indicated by the non-significant Cochran's Q test and MR-Egger intercept/MR-PRESSO *P*-values > 0.05 (Figure S1A and Table S10 in Supplementary materials). The leave-one-out analysis also confirmed these results (Figure S2A and Table S11 in Supplementary materials). These findings provide strong statistical evidence supporting the negative association between HDL-C levels and the risk of SUI.

To address the potential for reverse causality, we conducted reverse MR analysis. When SUI was used as the exposure instrument, seven IVs were identified (Table S12 in Supplementary materials). As shown in Figure 5, all five Mendelian randomization methods yielded *P*-values greater than 0.05, providing no evidence of reverse causality. Additionally, the heterogeneity and horizontal pleiotropy analysis of the reverse MR analysis was also deemed reliable (Figure S1B, Figure S2B and Table S13 in Supplementary materials).

## Discussion

This study comprehensively examined the association between HDL-C levels and SUI using data from NHANES and TSMR analysis. We found that higher HDL-C levels were consistently associated with a reduced risk of SUI, even after adjusting for factors like age, BMI, and metabolic comorbidities. This protective effect appeared to be more pronounced in younger individuals under 60 years and those with a BMI ≥ 25 kg/m². Additionally, TSMR analysis suggested a causal relationship, supporting HDL-C's protective effect against SUI.

A previous study indicated that women with HDL-C levels of 1.64 mmol/L (63.44 mg/dL) or higher had a 47.9% reduced risk of SUI 4. Although this was a single-center retrospective study, it suggested that higher HDL-C may provide protective effects against SUI. Similarly, our findings also demonstrate a negative association between HDL-C levels and the risk of developing SUI in women. Notably, our further research showed a negative correlation between HDL-C levels and the frequency of SUI. However, the MR analysis by Xiang et al. focused on lipid-glucose metabolism interactions, which identified a positive association between HDL-C and SUI in European women 11. Although our subgroup analysis also showed variable relationship between HDL-C and SUI among different races, there is still a significant protective effect among non-Hispanic whites. In addition, our MR analysis combined multiple European datasets, and strictly controlled for pleiotropy through Steiger filtering and sensitivity tests (MR-Egger, MR-PRESSO). By analyzing a large cross-sectional dataset and incorporating MR analysis, we demonstrated the role of HDL-C in the occurrence and progression of SUI.

The pathogenesis of SUI involves pelvic floor dysfunction, urethral sphincter dysfunction, and hormonal changes 30, 31. Lipid metabolism is increasingly recognized as playing a crucial role in the development of SUI. First, dyslipidemia often leads to excessive fat accumulation, which subsequently triggers obesity. Previous studies have demonstrated that obesity, particularly abdominal obesity, is closely related to the onset and severity of SUI 32. The accumulation of abdominal fat increases intra-abdominal pressure, which raises bladder stress and exacerbates detrusor instability. Secondly, animal studies have shown that prolonged exposure to hyperlipidemic conditions leads to epigenetic changes in female rats, resulting in altered gene and microRNA transcription profiles, and impairing the repair capacity of muscle-derived stem cells 33. This may weaken the pelvic floor's ability to recover, potentially contributing to the development of SUI. Meanwhile, our multivariate logistic model showed that LDL-C and TC had no association with SUI, but HDL-C stayed significant in all models. This may make our research clearer about the specific dyslipidemia for SUI.

Our TSMR analysis provides robust genetic evidence supporting a causal protective effect of HDL-C against SUI, with sensitivity analyses confirming minimal pleiotropy and heterogeneity. This aligns with HDL-C's pleiotropic roles in other systemic diseases, such as chronic kidney disease (CKD) 34 and chronic liver failure 35, where low HDL-C levels (< 30 mg/dL and < 17 mg/dL, respectively) predict adverse outcomes. Mechanistically, HDL-C may mitigate SUI risk through multiple pathways targeting pelvic floor integrity. Firstly, HDL-C suppresses the activity of matrix metalloproteinases (MMPs), particularly MMP-9 36, 37. This may reduce degradation of collagen and elastin in pelvic connective tissues, preserving the structural support of the urethra and bladder neck. Followed by anti-inflammatory and metabolic Regulation, by promoting endothelial nitric oxide synthase activation, HDL-C enhances nitric oxide bioavailability in pelvic microvasculature 38. This attenuates oxidative stress-induced endothelial dysfunction, improving blood flow to pelvic floor muscles and nerves critical for continence. Our subgroup analysis also demonstrated that the protective effect of HDL-C on SUI appeared to be more pronounced in those women with overweight or obesity. In obese individuals, chronic inflammation and insulin resistance can impair pelvic floor tissue repair and exacerbate endothelial damage 39. HDL-C's ability to mitigate collagen degradation and inflammation may counteract these effects, explaining its amplified protection in high-BMI populations. In addition, clinical studies have shown that bariatric surgery (such as laparoscopic sleeve gastrectomy and Roux-en-Y gastric bypass) can not only achieve sustained weight loss but also significantly increase HDL-C levels within 5 years after surgery 40, 41. This change is closely related to reduced visceral fat, enhanced insulin sensitivity 42, and inhibition of chronic inflammation 43. The increase in HDL-C not only enhances its inherent anti-inflammatory and antioxidant functions but also indirectly strengthens the stability of the urethral support structure by reversing pelvic microcirculatory disorders and inhibiting connective tissue degradation. At the same time, surgically induced weight loss directly reduces the mechanical load of intra-abdominal pressure on the bladder neck and urethra, thereby forming a synergistic effect with the metabolic protection provided by HDL-C, ultimately reducing the prevalence of SUI in obese patients. These studies not only confirm the core position of HDL-C in SUI protection, but also provide a metabolic-mechanical integrated treatment paradigm for clinical intervention of obesity-related SUI.

However, the protective effects of HDL-C are not universally linear. The “HDL-C paradox” 44 —where extreme elevations may diminish or reverse benefits—was observed in our unadjusted RCS analysis, with risk reduction plateauing at higher levels. This nonlinearity likely reflects confounding by metabolic comorbidities (e.g., diabetes, hypertension), as fully adjusted models restored a linear dose-response relationship. Similar associations, even U-shaped associations, have been reported for HDL-C-related cardiovascular mortality 45 and all-cause mortality 12, suggesting a shared biological threshold effect. These findings underscore the need for precision in HDL-C management. While elevating HDL-C may benefit most women with SUI—particularly those with obesity—extreme levels should be approached cautiously. Future studies must define optimal HDL-C targets for pelvic health and explore whether functional HDL properties (e.g., particle size, apolipoprotein composition) outweigh absolute levels in SUI prevention.

Our study has several strengths, including the use of a large, nationally representative sample from a large-scale cross-sectional analysis and the integration of dual evidence from MR, which enhances the robustness of our causal inference. Comprehensive adjustment for confounders and exploration of dose-response relationships further strengthen the reliability of our findings. However, several limitations should be acknowledged. First, the diagnosis of SUI relied solely on a single self-reported question in NHANES, which lacks the comprehensive clinical assessment. This approach may introduce misclassification bias, such as overestimation of prevalence due to subjective reporting. Future studies should prioritize datasets incorporating clinician physical examinations and multi-item questionnaires to enhance diagnostic specificity. Second, MR analysis relies on the strength and validity of genetic instruments, and potential pleiotropy may introduce bias into the results. Lastly, while our NHANES analysis is based on a U.S. population, the MR analysis data comes from individuals of European descent. Applying causal relationships derived from European populations to the U.S. population may not fully reflect the latter's specific characteristics, and the generalizability to other racial or geographic groups warrants further investigation.

## Conclusion

Through combined observational and Mendelian randomization analyses, we demonstrate that elevated HDL-C levels are causally associated with reduced SUI risk, with particularly strong protective effects observed in overweight/obese individuals. These robust findings suggest that maintaining optimal HDL-C levels may serve as an effective preventive strategy against SUI, especially in high-BMI populations. While our results highlight the therapeutic potential of HDL-C, future studies should further elucidate the underlying mechanisms and validate these associations across diverse demographic groups.

## Supplementary Material

Supplementary figures and tables.

## Figures and Tables

**Table 1:** Characteristics of study participants

Variable	Overall (n=18415)	No-SUI (n=10757)	SUI (n=7658)	*P-*value
Age, n (%)				
< 40 years	6013 (32.65)	4326 (40.22)	1687 (22.03)	< 0.001
40-59 years	6314 (34.29)	3165 (29.42)	3149 (41.12)	
≥ 60 years	6088 (33.06)	3266 (30.36)	2822 (36.85)	
Race, n (%)				
Non-Hispanic White	8415 (45.70)	4512 (41.94)	3903 (50.97)	< 0.001
Mexican American	2875 (15.61)	1543 (14.34)	1332 (17.39)	
Other Hispanic	1611 (8.75)	945 (8.78)	666 (8.70)	
Non-Hispanic Black	3851 (20.91)	2690 (25.01)	1161 (15.16)	
Other Race	1663 (9.03)	1067 (9.92)	596 (7.78)	
Education, n (%)				
Less than high school	4083 (22.17)	2259 (21.00)	1824 (23.82)	< 0.001
High school	4118 (22.36)	2367 (22.00)	1751 (22.86)	
Greater than high school	10214 (55.47)	6131 (57.00)	4083 (53.32)	
Marital status, n (%)				
Married/living with partner	10053 (54.59)	5560 (51.69)	4493 (58.67)	< 0.001
Living alone	8362 (45.41)	5197 (48.31)	3165 (41.33)	
PIR, n (%)				
≥ 2	9699 (52.67)	5643 (52.46)	4056 (52.96)	0.508
< 2	8716 (47.33)	5114 (47.54)	3602 (47.04)	
BMI, n (%)				
< 25 kg/m^2^	5655 (30.71)	3843 (35.73)	1812 (23.66)	< 0.001
25 - 30 kg/m2	5262 (28.57)	3042 (28.28)	2220 (28.99)	
≥ 30 kg/m^2^	7498 (40.72)	3872 (36.00)	3626 (47.35)	
Hypertension, n (%)				
No	11816 (64.17)	7377 (68.58)	4439 (57.97)	< 0.001
Yes	6599 (35.83)	3380 (31.42)	3219 (42.03)	
Diabetes, n (%)				
No	16213 (88.04)	9703 (90.20)	6510 (85.01)	< 0.001
Yes	2202 (11.96)	1054 (9.80)	1148 (14.99)	
Vigorous recreational activities, n (%)				
No	14596 (79.26)	8250 (76.69)	6346 (82.87)	< 0.001
Yes	3819 (20.74)	2507 (23.31)	1312 (17.13)	
Smoke, n (%)				
Never	11543 (62.68)	7070 (65.72)	4473 (58.41)	< 0.001
Former	3594 (19.52)	1887 (17.54)	1707 (22.29)	
Current	3278 (17.80)	1800 (16.73)	1478 (19.30)	
Alcohol, n (%)				
No	6417 (34.85)	3854 (35.83)	2563 (33.47)	0.001
Yes	11998 (65.15)	6903 (64.17)	5095 (66.53)	
CHD, n (%)				
No	17948 (97.46)	10538 (97.96)	7410 (96.76)	< 0.001
Yes	467 (2.54)	219 (2.04)	248 (3.24)	
Stroke, n (%)				
No	17741 (96.34)	10453 (97.17)	7288 (95.17)	< 0.001
Yes	674 (3.66)	304 (2.83)	370 (4.83)	
Cancer/malignancy, n (%)				
No	16581 (90.04)	9856 (91.62)	6725 (87.82)	< 0.001
Yes	1834 (9.96)	901 (8.38)	933 (12.18)	
FBG (mean (SD)), mg/dL	106.20 (34.43)	103.87 (32.86)	109.46 (36.27)	< 0.001
INS (mean (SD)), uU/mL	13.17 (15.82)	12.26 (15.15)	14.44 (16.63)	< 0.001
ALB (mean (SD)), g/dL	4.14 (0.32)	4.15 (0.32)	4.12 (0.32)	< 0.001
ALT (mean (SD)), U/L	21.13 (19.73)	20.46 (22.59)	22.07 (14.76)	< 0.001
AST (mean (SD)), U/L	23.24 (13.71)	22.88 (14.15)	23.74 (13.06)	< 0.001
BUN (mean (SD)), mg/dL	13.03 (5.82)	12.70 (5.78)	13.48 (5.85)	< 0.001
SCr (mean (SD)), mg/dL	0.80 (0.37)	0.80 (0.42)	0.80 (0.28)	0.885
TBil (mean (SD)), mg/dL	0.59 (0.29)	0.60 (0.28)	0.58 (0.29)	0.002
TG (mean (SD)), mg/dL	117.82 (93.40)	109.64 (95.38)	129.27 (89.32)	< 0.001
Total Cholesterol (mean (SD)), mg/dL	196.62 (41.05)	194.312(40.80)	199.87 (41.19)	< 0.001
LDL-C (mean (SD)), mg/dL	113.87 (35.51)	111.99 (35.24)	116.50 (35.73)	<0.001
HDL-C (mean (SD)), mg/dL	57.75 (16.41)	58.77 (16.51)	56.31 (16.17)	< 0.001
HDL-C quartiles, n (%)				
Q1	4824 (26.20)	2566 (23.85)	2258 (29.49)	< 0.001
Q2	4833 (26.24)	2759 (25.65)	2074 (27.08)	
Q3	4375 (23.76)	2633 (24.48)	1742 (22.75)	
Q4	4383 (23.80)	2799 (26.02)	1584 (20.68)	

HDL-C: high density lipoprotein cholesterol; SUI: stress urinary incontinence; PIR: poverty-income ratio; BMI: body mass index; CHD: coronary heart disease; FBG: fasting blood glucose; INS: insulin; ALB: albumin; ALT: alanine aminotransferase; AST: aspartate aminotransferase; BUN: blood urea nitrogen; SCr: serum creatinine; TBil: total Bilirubin; LDL-C: low density lipoprotein cholesterol; SD: standard deviation. *P*-value < 0.05 was considered significant.

**Table 2:** Associations between HDL-C and SUI

Variables	Model 1		Model 2		Model 3	
	OR (95% CI)	*P*-value	OR (95% CI)	*P*-value	OR (95% CI)	*P*-value
Continuous	0.992 (0.989 - 0.994)	< 0.001	0.988 (0.986 - 0.991)	< 0.001	0.995 (0.992 - 0.998)	< 0.001
Categories						
< 67mg/dL	ref		ref		ref	
≥ 67mg/dL	0.763 (0.698 - 0.835)	< 0.001	0.684 (0.624 - 0.749)	< 0.001	0.827 (0.747 - 0.917)	< 0.001
Quartiles						
Q1	ref		ref		ref	
Q2	0.870 (0.784 - 0.965)	0.009	0.852 (0.763 - 0.952)	0.005	0.950 (0.847-1.066)	0.380
Q3	0.726 (0.653 - 0.808)	< 0.001	0.703 (0.630 - 0.785)	< 0.001	0.852 (0.759-0.956)	0.007
Q4	0.645 (0.578 - 0.720)	< 0.001	0.563 (0.502 - 0.632)	< 0.001	0.749 (0.652-0.859)	< 0.001
*P* for trend	< 0.001		< 0.001		< 0.001	

HDL-C: high-density lipoprotein cholesterol; SUI: stress urinary incontinence; OR: odds ratio; CI: confidence intervals. Model 1 was an unadjusted crude model. Model 2 adjusted for age, race, marital status, PIR, and education. Model 3 adjusted for age, race, marital status, PIR, education, BMI, hypertension, diabetes, alcohol use, smoke, vigorous recreational activities, coronary heart disease, stroke, cancer/malignancy, TC, ALT, AST, ALB, SCr, BUN, and TBil. *P*-value < 0.05 was considered significant.

## Abbreviations

SUI: stress urinary incontinence
HDL-C: high-density lipoprotein cholesterol
MR: mendelian randomization
NHANES: national Health and Nutrition Examination Survey
TSMR: two-sample mendelian randomization
MEC: mobile Examination Center
GWAS: genome-wide association study
CHD: coronary heart disease
TC: total cholesterol
ALT: alanine aminotransferase
AST: aspartate aminotransferase
ALB: albumin
SCr: serum creatinine
BUN: blood urea nitrogen
TBil: total bilirubin
SNPs: single nucleotide polymorphisms
IVs: instrumental variables
RCS: restricted cubic spline
IVW: inverse variance weighting
OR: odds ratio
MMPs: Matrix metalloproteinases
CKD: chronic kidney disease
