# Supplementary material for: Protective Role of High-Density Lipoprotein Cholesterol in Stress Urinary Incontinence with Special Emphasis on Overweight/Obese Individuals
Source: Int J Med Sci. 2025 Jul 11;22(13):3304–15. doi: 10.7150/ijms.116324 (PMC12320798; doi:10.7150/ijms.116324)
Supplement: Supplementary file 1 — Supplementary figures and tables. [file ijmsv22p3304s1.pdf]

Supplementary materials

Supplementary figures

Figure S1. (A) Funnel plot of the causal relationship between HDL-C and SUI; (B) Funnel plot of the reverse causal relationship between HDL-C and SUI.

Figure S2. (A) Mendelian randomization leave-one-out sensitivity analysis graph for HDL-C on SUI; (B)Mendelian randomization leave-one-out sensitivity analysis graph for SUI on HDL-C.

Supplementary tables

Table S1. Weighted characteristics of study participants.

Table S2. Associations between TC, LDL-C, and the risk of SUI.

Table S3. Subgroup analysis of HDL-C categories and SUI.

Table S4. Subgroup analysis of HDL-C quartiles and SUI.

Table S5. Sensitivity analysis of association between HDL-C and SUI adjusted for data release cycle.

Table S6. Sensitivity analysis of association between HDL-C and SUI adjusted for blood sampling time.

Table S7. Sensitivity analysis of association between HDL-C and SUI adjusted for LDL-C.

Table S8. Sensitivity analysis of the association between HDL-C and different degrees of SUI.

Table S9. Significant SNPs with genome-wide associations ($P < 5\times10^{-8}$) for HDL-C.

Table S10. Sensitivity analysis in MR analysis.

Table S11. MR leave−one−out sensitivity analysis for HDL-C on SUI.

Table S12. Significant SNPs with genome-wide associations ($P < 1\times10^{-6}$) for SUI.

Table S13. Causality and sensitivity analysis of SUI and HDL-C in reverse two-sample MR analysis.

Supplementary figures

Figure S1. (A) Funnel plot of the causal relationship between HDL-C and SUI; (B) Funnel plot of the reverse causal relationship between HDL-C and SUI.

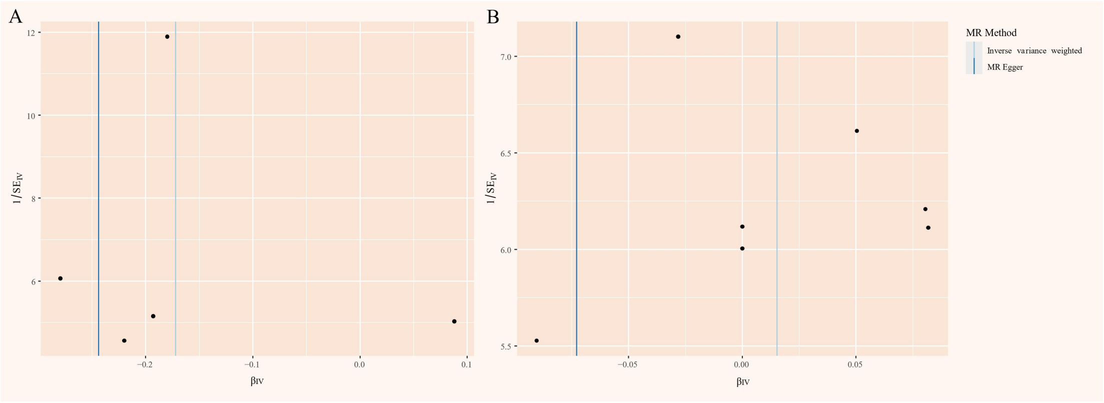

HDL-C: high density lipoprotein cholesterol; SUI: stress urinary incontinence; MR: mendelian randomization; β_{IV}: effect size of instrumental variables; SE_{IV}: the standard error of the instrumental variable effect size.

Figure S2. (A) Mendelian randomization leave-one-out sensitivity analysis graph for HDL-C on SUI; (B)Mendelian randomization leave-one-out sensitivity analysis graph for SUI on HDL-C.

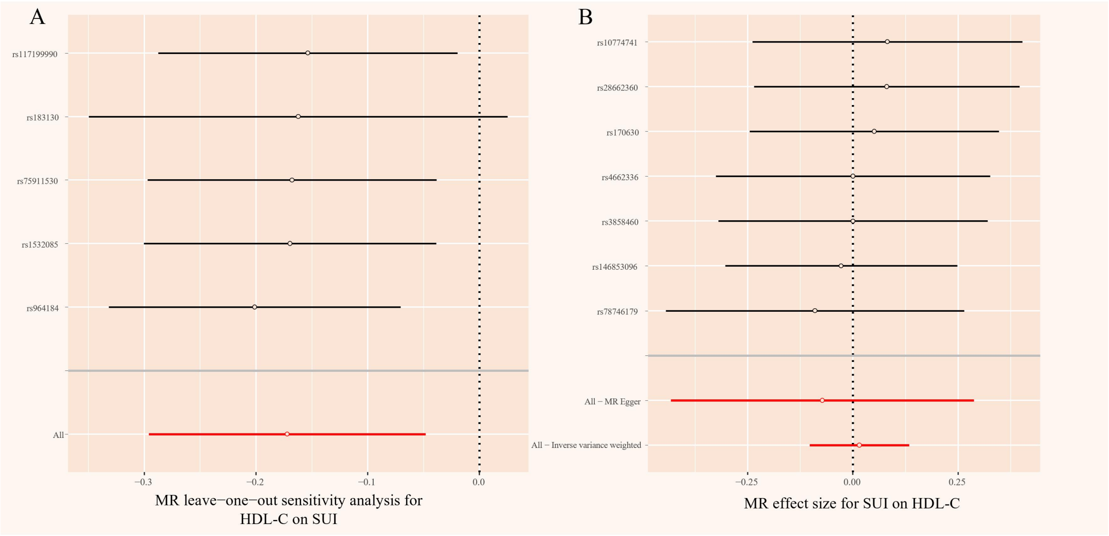

HDL-C: high density lipoprotein cholesterol; SUI: stress urinary incontinence; MR: mendelian randomization.

Supplementary tables

Table S1. Weighted characteristics of study participants.

Variable	Overall (n=85864847)	No-SUI (n=49046999)	SUI (n=36817847)	*P* -value
Age, n(%)				
< 40 years	30029943.66 (34.97)	21439900.15 (43.71)	8590043.51 (23.33)	<0.001
40-59 years	33061348.16 (38.50)	16193410.06 (33.02)	16867938.09 (45.81)	
≥ 60 years	22773554.69 (26.52)	11413689.01 (23.27)	11359865.69 (30.85)	
Race, n(%)				
Non-Hispanic White	60490922.86 (70.45)	32905510.43 (67.09)	27585412.43 (74.92)	<0.001
Mexican American	6087963.35 (7.09)	3384700.01 (6.90)	2703263.34 (7.34)	
Other Hispanic	4433546.37 (5.16)	2745731.52 (5.60)	1687814.85 (4.58)	
Non-Hispanic Black	9306466.90 (10.84)	6560314.69 (13.38)	2746152.21 (7.46)	
Other Race	5545947.02 (6.46)	3450742.58 (7.04)	2095204.45 (5.69)	
Education, n(%)				
Less than high school	11993150.06 (13.97)	6476870.81 (13.21)	5516279.25 (14.98)	0.002
High school	19544741.80 (22.76)	10942013.31 (22.31)	8602728.49 (23.37)	
Greater than high school	54326954.65 (63.27)	31628115.10 (64.49)	22698839.55 (61.65)	
Marital status, n(%)				
Married/living with partner	52019824.35 (60.58)	27994335.71 (57.08)	24025488.64 (65.26)	<0.001
Living alone	33845022.16 (39.42)	21052663.52 (42.92)	12792358.65 (34.74)	
PIR, n(%)				
≥ 2	55867031.50 (65.06)	31468745.90 (64.16)	24398285.60 (66.27)	0.007
< 2	29997815.01 (34.94)	17578253.33 (35.84)	12419561.68 (33.73)	
BMI, n(%)				
< 25 kg/m^2	29607094.38 (34.48)	19756313.04 (40.28)	9850781.34 (26.76)	<0.001

25-30 kg/m2	24083737.67 (28.05)	13519857.13 (27.57)	10563880.55 (28.69)	
≥ 30 kg/m^2	32174014.46 (37.47)	15770829.06 (32.15)	16403185.40 (44.55)	
Hypertension, n(%)				
No	59175144.13 (68.92)	36241299.04 (73.89)	22933845.09 (62.29)	<0.001
Yes	26689702.38 (31.08)	12805700.18 (26.11)	13884002.20 (37.71)	
Diabetes, n(%)				
No	78438450.88 (91.35)	45747500.99 (93.27)	32690949.89 (88.79)	<0.001
Yes	7426395.63 (8.65)	3299498.24 (6.73)	4126897.39 (11.21)	
Vigorous recreational activities, n(%)				
No	63861450.98 (74.37)	35017078.56 (71.39)	28844372.42 (78.34)	<0.001
Yes	22003395.53 (25.63)	14029920.67 (28.61)	7973474.86 (21.66)	
Smoke, n(%)				
No	51462252.33 (59.93)	30633871.27 (62.46)	20828381.06 (56.57)	<0.001
Former	18395428.49 (21.42)	9547989.78 (19.47)	8847438.71 (24.03)	
Current	16007165.70 (18.64)	8865138.18 (18.07)	7142027.52 (19.40)	
Alcohol, n(%)				
No	23733522.40 (27.64)	13801482.91 (28.14)	9932039.49 (26.98)	0.136
Yes	62131324.11 (72.36)	35245516.32 (71.86)	26885807.80 (73.02)	
CHD, n(%)				
No	83906652.14 (97.72)	48197935.03 (98.27)	35708717.11 (96.99)	<0.001
Yes	1958194.37 (2.28)	849064.20 (1.73)	1109130.18 (3.01)	
Stroke, n(%)				
No	83283795.23 (96.99)	47961817.90 (97.79)	35321977.33 (95.94)	<0.001
Yes	2581051.28 (3.01)	1085181.33 (2.21)	1495869.96 (4.06)	
Cancer/malignancy, n(%)				
No	76378459.77	44456076.59	31922383.18	<0.00

	(88.95)	(90.64)	(86.70)	1
Yes	9486386.74 (11.05)	4590922.63 (9.36)	4895464.10 (13.30)	
FBG (mean (SD)), mg/dL	102.825 (28.117)	100.640 (26.117)	105.678 (30.300)	<0.001
INS (mean (SD)), uU/mL	12.141 (14.816)	11.413 (14.406)	13.096 (15.285)	<0.001
ALB (mean (SD)), g/dL	4.172 (0.313)	4.191 (0.312)	4.148 (0.313)	<0.001
ALT (mean (SD)), U/L	21.024 (20.972)	20.435 (24.932)	21.808 (14.021)	<0.001
AST (mean (SD)), U/L	22.992 (12.833)	22.691 (12.833)	23.394 (12.822)	0.006
BUN (mean (SD)), mg/dL	12.872 (5.350)	12.491 (5.243)	13.380 (5.449)	<0.001
SCr (mean (SD)), mg/dL	0.787 (0.301)	0.784 (0.344)	0.792 (0.232)	0.087
TBil (mean (SD)), mg/dL	0.593 (0.273)	0.602 (0.271)	0.582 (0.276)	<0.001
TG (mean (SD)), mg/dL	116.529 (93.194)	108.377 (90.360)	127.231 (95.752)	<0.001
Total Cholesterol (mean (SD)), mg/dL	197.341 (40.657)	194.611 (40.286)	200.977 (40.867)	<0.001
LDL-C (mean (SD)), mg/dL	114.236 (35.099)	112.204 (34.995)	116.906 (35.062)	<0.001
HDL-C (mean (SD)), mg/dL	58.786 (16.788)	59.759 (16.670)	57.491 (16.859)	<0.001
HDL-C quartiles, n(%)				
Q1	20682406.48 (24.09)	10657796.56 (21.73)	10024609.92 (27.23)	<0.001
Q2	21780386.28 (25.37)	11981091.90 (24.43)	9799294.38 (26.62)	
Q3	21423453.59 (24.95)	12728673.50 (25.95)	8694780.09 (23.62)	
Q4	21978600.17 (25.60)	13679437.26 (27.89)	8299162.90 (22.54)	

HDL-C: high density lipoprotein cholesterol; SUI: stress urinary incontinence; PIR: poverty-income ratio; BMI: body mass index; CHD: coronary heart disease; FBG: fasting blood glucose; INS: insulin; ALB: albumin; ALT: alanine aminotransferase; AST: aspartate aminotransferase; BUN: blood urea nitrogen; SCr: serum creatinine; TBil: total bilirubin; LDL-C: low density lipoprotein cholesterol; SD: standard deviation.

P-value < 0.05 was considered significant.

Table S2. Associations between TC, LDL-C, and the risk of SUI.

Variables	Model 1		Model 2		Model 3	
	OR (95% CI)	*P*-value	OR (95% CI)	*P*-value	OR (95% CI)	*P*-value
TC	1.004 (1.003 - 1.005)	< 0.001	1.001 (1.000 - 1.002)	0.199	1.001 (1.000 - 1.002)	0.140
LDL-C	1.004 (1.002 - 1.006)	< 0.001	1.001 (0.999 - 1.003)	0.190	1.002 (0.998 - 1.006)	0.271

TC: total cholesterol; LDL-C: low density lipoprotein cholesterol; SUI: stress urinary incontinence; OR: odds ratio; CI: confidence intervals.

Model 1 was an unadjusted crude model.

Model 2 adjusted for age, race, marital status, PIR, and education.

Model 3 adjusted for age, race, marital status, PIR, education, BMI, hypertension, diabetes, alcohol use, smoke,vigorous recreational activities, coronary heart disease, stroke, cancer/malignancy, ALT, AST, ALB, SCr, BUN, and TBil.

P-value < 0.05 was considered significant.

Table S3. Subgroup analysis of HDL-C categories and SUI.

Subgroup	OR (95% CI)	*P*-value
HDL-C ≥ 67mg/dL	Ref	
Age, n(%)		
< 40 years	1.337 (1.095 - 1.634)	0.005
40-59 years	1.254 (1.048 - 1.500)	0.014
≥ 60 years	1.128 (0.947 - 1.343)	0.175
Race, n(%)		
Non-Hispanic White	1.226 (1.074 - 1.399)	0.003
Mexican American	1.529 (1.186 - 1.971)	0.001
Other Hispanic	1.024 (0.731 - 1.436)	0.888
Non-Hispanic Black	1.030 (0.841 - 1.261)	0.771
Other Race	1.327 (0.956 - 1.842)	0.090
Education, n(%)		
Less than high school	1.055 (0.852 - 1.306)	0.623
High school	1.288 (1.016 - 1.632)	0.037
Greater than high school	1.224 (1.079 - 1.388)	0.002
Marital status, n(%)		
Married/living with partner	1.224 (1.057 - 1.418)	0.007
Living alone	1.200 (1.033 - 1.394)	0.018
PIR, n(%)		
≥ 2	1.220 (1.067 - 1.395)	0.004
< 2	1.219 (1.025 - 1.450)	0.025
BMI, n(%)		
< 25 kg/m^2	0.985 (0.831 - 1.168)	0.863
25-30 kg/m^2	1.367 (1.151 - 1.623)	0.001
≥ 30 kg/m^2	1.486 (1.247 - 1.770)	0.000
Hypertension, n(%)		
Yes	1.169 (0.983 - 1.389)	0.076
No	1.238 (1.096 - 1.399)	0.001
Diabetes, n(%)		
Yes	1.478 (1.029 - 2.124)	0.035
No	1.196 (1.073 - 1.331)	0.001
Smoke, n(%)		
No	1.231 (1.096 - 1.383)	0.001
Former	1.135 (0.911 - 1.414)	0.255
Current	1.299 (1.011 - 1.671)	0.041
Alcohol, n(%)		
Yes	1.198 (1.061 - 1.352)	0.004
No	1.226 (1.020 - 1.474)	0.031
Vigorous recreational activities, n(%)		
Yes	1.178 (0.974 - 1.425)	0.090
No	1.224 (1.088 - 1.377)	0.001
Coronary heart disease, n(%)		

Yes	0.595 (0.277 - 1.275)	0.177
No	1.224 (1.103 - 1.358)	0.000
Stroke, n(%)		
Yes	0.760 (0.448 - 1.287)	0.303
No	1.229 (1.105 - 1.368)	0.000
Cancer/malignancy, n(%)		
Yes	1.114 (0.843 - 1.472)	0.444
No	1.216 (1.087 - 1.361)	0.001

HDL-C: high density lipoprotein cholesterol; SUI: stress urinary incontinence; PIR: poverty-income ratio; BMI: body mass index; OR; odds ratio; CI: confidence intervals.

P-value < 0.05 was considered significant.

Table S4. Subgroup analysis of HDL-C quartiles and SUI.

Subgroup	Q1	Q2		Q3		Q4	
		OR (95% CI)	*P*-value	OR (95% CI)	*P*-value	OR (95% CI)	*P*-value
Age, n(%)							
< 40 years	-	0.927 (0.760 - 1.131)	0.452	0.805 (0.655 - 0.990)	0.040	0.634 (0.499 - 0.805)	< 0.001
40-59 years	-	0.949 (0.780 - 1.155)	0.599	0.849 (0.711 - 1.014)	0.071	0.727 (0.592 - 0.893)	0.003
≥ 60 years	-	0.977 (0.798 - 1.197)	0.822	0.872 (0.701 - 1.085)	0.217	0.822 (0.655 - 1.032)	0.091
Race, n(%)							
Non-Hispanic White	-	0.956 (0.816 - 1.121)	0.581	0.837 (0.718 - 0.976)	0.024	0.733 (0.611 - 0.880)	0.001
Mexican American	-	1.072 (0.853 - 1.347)	0.547	0.853 (0.664 - 1.098)	0.214	0.614 (0.462 - 0.817)	0.001
Other Hispanic	-	1.055 (0.778 - 1.431)	0.728	0.999 (0.688 - 1.451)	0.995	0.981 (0.680 - 1.415)	0.917
Non-Hispanic Black	-	0.816 (0.660 - 1.010)	0.062	0.792 (0.645 - 0.973)	0.027	0.811 (0.631 - 1.043)	0.101
Other Race	-	0.866 (0.572 - 1.312)	0.494	0.971 (0.678 - 1.392)	0.872	0.703 (0.464 - 1.066)	0.096
Education, n(%)							
Less than high school	-	1.025 (0.833 - 1.261)	0.816	0.914 (0.726 - 1.151)	0.441	0.939 (0.731 - 1.206)	0.618
High school	-	0.999 (0.798 - 1.250)	0.992	0.925 (0.744 - 1.151)	0.481	0.742 (0.556 - 0.991)	0.043
Greater than high school	-	0.900 (0.767 - 1.057)	0.197	0.801 (0.683 - 0.940)	0.007	0.698 (0.583 - 0.836)	< 0.001
Marital status, n(%)							
Married/living with	-	0.978	0.7	0.885	0.1	0.754	0.0

partner		(0.848 - 1.129)	62	(0.757 - 1.036)	27	(0.622 - 0.913)	04
Living alone	-	0.899 (0.749 - 1.080)	0.254	0.796 (0.674 - 0.941)	0.008	0.729 (0.599 - 0.888)	0.002
PIR, n(%)							
≥ 2	-	0.969 (0.819 - 1.146)	0.712	0.877 (0.756 - 1.018)	0.083	0.750 (0.626 - 0.899)	0.002
< 2	-	0.933 (0.816 - 1.068)	0.313	0.817 (0.684 - 0.976)	0.026	0.742 (0.599 - 0.918)	0.006
BMI, n(%)							
< 25 kg/m^2	-	0.706 (0.535 - 0.933)	0.015	0.771 (0.589 - 1.008)	0.058	0.773 (0.597 - 1.001)	0.051
25-30 kg/m^2	-	1.094 (0.883 - 1.356)	0.410	0.913 (0.735 - 1.133)	0.404	0.696 (0.554 - 0.875)	0.002
≥ 30 kg/m^2	-	1.010 (0.853 - 1.195)	0.910	0.858 (0.699 - 1.053)	0.141	0.666 (0.537 - 0.827)	< 0.001
Hypertension, n(%)							
Yes	-	1.006 (0.839 - 1.207)	0.948	0.990 (0.818 - 1.197)	0.915	0.858 (0.689 - 1.068)	0.168
No	-	0.919 (0.794 - 1.065)	0.259	0.789 (0.678 - 0.919)	0.003	0.689 (0.582 - 0.816)	< 0.001
Diabetes, n(%)							
Yes	-	0.960 (0.709 - 1.301)	0.793	0.910 (0.647 - 1.279)	0.583	0.644 (0.434 - 0.955)	0.029
No	-	0.949 (0.837 - 1.075)	0.407	0.846 (0.750 - 0.955)	0.007	0.752 (0.650 - 0.870)	< 0.001
Smoke, n(%)							
No	-	0.897 (0.768 - 1.048)	0.169	0.865 (0.741 - 1.008)	0.064	0.702 (0.598 - 0.824)	< 0.001
Former	-	1.002 (0.780 - 1.288)	0.986	0.932 (0.738 - 1.178)	0.552	0.894 (0.676 - 1.181)	0.426

Current	-	1.057 (0.819 - 1.364)	0.667	0.688 (0.530 - 0.892)	0.005	0.702 (0.513 - 0.960)	0.027
Alcohol, n(%)							
Yes	-	0.917 (0.797 - 1.055)	0.225	0.818 (0.713 - 0.939)	0.005	0.738 (0.627 - 0.867)	< 0.001
No	-	1.031 (0.862 - 1.233)	0.736	0.941 (0.774 - 1.143)	0.536	0.769 (0.610 - 0.971)	0.027
Vigorous recreational activities, n(%)							
Yes	-	0.894 (0.682 - 1.171)	0.413	0.789 (0.607 - 1.025)	0.075	0.711 (0.546 - 0.928)	0.012
No	-	0.961 (0.851 - 1.085)	0.518	0.870 (0.765 - 0.989)	0.033	0.754 (0.650 - 0.874)	< 0.001
Coronary heart disease, n(%)							
Yes	-	1.670 (0.885 - 3.149)	0.111	0.999 (0.506 - 1.971)	0.997	2.189 (0.971 - 4.935)	0.059
No	-	0.935 (0.833 - 1.050)	0.254	0.845 (0.752 - 0.949)	0.005	0.731 (0.635 - 0.841)	< 0.001
Stroke, n(%)							
Yes	-	0.743 (0.416 - 1.327)	0.311	0.565 (0.305 - 1.047)	0.069	0.926 (0.482 - 1.778)	0.815
No	-	0.957 (0.852 - 1.075)	0.452	0.861 (0.763 - 0.972)	0.016	0.742 (0.641 - 0.859)	< 0.001
Cancer/malignancy, n(%)							
Yes	-	0.827 (0.572 - 1.195)	0.309	0.760 (0.529 - 1.093)	0.137	0.734 (0.523 - 1.028)	0.072
No	-	0.963 (0.856 - 1.084)	0.532	0.866 (0.767 - 0.977)	0.020	0.755 (0.651 - 0.875)	< 0.001

HDL-C: high density lipoprotein cholesterol; SUI: stress urinary incontinence; PIR: poverty-income ratio; BMI: body mass index; OR: odds ratio; CI: confidence intervals.

P-value < 0.05 was considered significant.

Table S5. Sensitivity analysis of association between HDL-C and SUI adjusted for data release cycle.

Variables	OR (95% CI)	*P*-value
Continuous	0.995 (0.992-0.998)	0.001
Categories		
< 67mg/dL	ref	
≥ 67mg/dL	0.829 (0.748-0.918)	< 0.001
Quartiles		
Q1	ref	
Q2	0.951 (0.848-1.067)	0.388
Q3	0.853 (0.759-0.958)	0.008
Q4	0.750 (0.653-0.861)	< 0.001
P for trend	< 0.001	

HDL-C: high density lipoprotein cholesterol; SUI: stress urinary incontinence; OR: odds ratio; CI: confidence intervals.

Model was adjusted for age, race, marital status, PIR, education, BMI, hypertension, diabetes, alcohol use, smoke,vigorous recreational activities, coronary heart disease, stroke, cancer/malignancy, TC, ALT, AST, ALB, SCr, BUN, TBil and data release cycle.

P-value < 0.05 was considered significant.

Table S6. Sensitivity analysis of association between HDL-C and SUI adjusted for blood sampling time.

Variables	OR (95% CI)	*P*-value
Continuous	0.995 (0.992 - 0.998)	0.001
Binary categories		
< 67mg/dL	ref	
≥ 67mg/dL	0.828 (0.748 - 0.916)	< 0.001
Four categories		
Q1	ref	
Q2	0.950 (0.847 - 1.065)	0.374
Q3	0.852 (0.758 - 0.957)	0.007
Q4	0.748 (0.652 - 0.858)	< 0.001
P for trend	< 0.001	

HDL-C: high density lipoprotein cholesterol; SUI: stress urinary incontinence; OR: odds ratio; CI: confidence intervals.

Model was adjusted for age, race, marital status, PIR, education, BMI, hypertension, diabetes, alcohol use, smoke,vigorous recreational activities, coronary heart disease, stroke, cancer/malignancy, TC, ALT, AST, ALB, SCr, BUN, TBil and blood sampling time.

P-value < 0.05 was considered significant.

Table S7 Sensitivity analysis of association between HDL-C and SUI adjusted for LDL-C.

Variables	OR (95% CI)	*P*-value
Continuous	0.992 (0.986 - 0.997)	0.005
Binary categories		
< 67mg/dL	ref	
≥ 67mg/dL	0.855 (0.718 - 1.017)	0.076
Four categories		
Q1	ref	
Q2	0.976 (0.822 - 1.158)	0.779
Q3	0.818 (0.681 - 0.982)	0.032
Q4	0.727 (0.580 - 0.913)	0.007
P for trend	0.003	

HDL-C: high density lipoprotein cholesterol; SUI: stress urinary incontinence; LDL-C: low density lipoprotein cholesterol; OR: odds ratio; CI: confidence intervals.

Model was adjusted for age, race, marital status, PIR, education, BMI, hypertension, diabetes, alcohol use, smoke,vigorous recreational activities, coronary heart disease, stroke, cancer/malignancy, TC, ALT, AST, ALB, SCr, BUN, TBil and LDL-C.

P-value < 0.05 was considered significant.

Table S8. Sensitivity analysis of the association between HDL-C and different degrees of SUI.

Variables	Mild SUI		Moderate SUI		Severe SUI	
	OR (95% CI)	*P*-value	OR (95% CI)	*P*-value	OR (95% CI)	*P*-value
Continuous	0.994 (0.991 - 0.998)	0.001	0.990 (0.985 - 0.995)	< 0.001	0.991 (0.984 - 0.999)	0.023
Categories						
< 67mg/dL	ref		ref		ref	
≥ 67mg/dL	0.831 (0.741 - 0.931)	0.002	0.713 (0.582 - 0.873)	0.001	0.626 (0.497 - 0.787)	< 0.001
Quartiles						
Q1	ref		ref		ref	
Q2	0.959 (0.842 - 1.093)	0.531	0.859 (0.702 - 1.052)	0.141	0.969 (0.776 - 1.212)	0.784
Q3	0.846 (0.745 - 0.960)	0.010	0.723 (0.572 - 0.913)	0.007	0.876 (0.683 - 1.124)	0.295
Q4	0.750 (0.647 - 0.870)	< 0.001	0.598 (0.472 - 0.756)	< 0.001	0.588 (0.446 - 0.775)	< 0.001
P for trend	< 0.001		< 0.001		< 0.001	

HDL-C: high density lipoprotein cholesterol; SUI: stress urinary incontinence; OR: odds ratio; CI: confidence intervals.

All model were adjusted for age, race, marital status, PIR, education, BMI, hypertension, diabetes, alcohol use, smoke,vigorous recreational activities, coronary heart disease, stroke, cancer/malignancy, TC, ALT, AST, ALB, SCr, BUN, TBil.

P-value < 0.05 was considered significant.

Table S9. Significant SNPs with genome-wide associations (*P* <5×10^{-8}) for HDL-C.

SNP	Chromosome	Position	Effect Allele	Other Allele	β	Standard Error	Effector Allele Frequency	*P*-value	Samplesize	R2	F	Steiger direction	Steiger *P*
rs117199990	8	19963405	T	C	0.19	0.02	0.1302	6.22E-14	9796	0.009128841	90.23157411	TRUE	4.38E-19
rs1532085	15	58391167	G	A	-0.1	0.02	0.6352	1.64E-11	9796	0.002545566	24.99489588	TRUE	2.83E-06
rs183130	16	56957451	T	C	0.24	0.02	0.2932	3.22E-54	9796	0.014486922	143.9706002	TRUE	1.93E-29
rs75911530	16	57015225	A	G	-0.25	0.04	0.0278	1.65E-08	9796	0.003971759	39.05452481	TRUE	3.61E-09
rs964184	11	116778201	C	G	0.14	0.02	0.838	2.00E-10	9796	0.004977146	48.98999592	TRUE	1.48E-11

HDL-C: high density lipoprotein cholesterol; SNP: single nucleotide polymorphism.

Table S10. Sensitivity analysis in MR analysis.

Exposure	Outcome	Method	Cochran's Q statistic (heterogeneity *P*)	MR Egger intercept(pleiotropy *P*)	MR PRESSO *P*
HDL-C	SUI	MR Egger	0.565924841	0.710369767	0.767
		IVW	0.699366476	0.710369767	0.767

HDL-C: high density lipoprotein cholesterol; SUI: stress urinary incontinence; MR: mendelian randomization; IVW: inverse variance weighting.

Table S11. MR leave−one−out sensitivity analysis for HDL-C on SUI.

Exposure	Outcome	SNP	β	Standard Error	*P* -value
HDL-C	SUI	rs117199990	-0.153702924	0.068353874	0.024535719
		rs1532085	-0.169656623	0.066781493	0.011070244
		rs183130	-0.162245977	0.095615968	0.08972498
		rs75911530	-0.167799754	0.06594551	0.010942765
		rs964184	-0.201253826	0.066591978	0.002509503
		All	-0.172122207	0.063148074	0.006416682

HDL-C: high density lipoprotein cholesterol; SUI: stress urinary incontinence; MR: mendelian randomization; SNP: single nucleotide polymorphism.

Table S12. Significant SNPs with genome-wide associations (*P* <1×10^{-6}) for SUI.

SNP	Chromosome	Position	Effect Allele	Other Allele	β	Standard Error	Effector Allele Frequency	*P*-value	Samplesize	R2	F	Steiger direction	Steiger *P*
rs10774741	12	114666585	A	G	-0.122244884	0.019692709	0.361999362	5.38E-10	217598	0.000177059	38.53421068	TRUE	0.424221421424
rs146853096	12	20686482	C	T	0.355069854	0.072000455	0.018425883	8.16E-07	217598	0.000111752	24.31941551	TRUE	0.407806467
rs170630	1	7906702	G	A	0.198410366	0.039325289	0.062918281	4.53E-07	217598	0.000116971	25.45545589	TRUE	0.470930509
rs28662360	2	28208920	G	A	-0.124164569	0.022819115	0.780375604	5.29E-08	217598	0.000136045	29.60695694	TRUE	0.522114292
rs3858460	11	32492447	T	C	-0.122350735	0.023723469	0.199513593	2.50E-07	217598	0.000122222	26.59823366	TRUE	0.284498567
rs4662336	2	144266448	T	G	-0.120088671	0.021816596	0.732766566	3.70E-08	217598	0.000139224	30.29884844	TRUE	0.253331625
rs75239609	12	24552189	T	C	0.218816199	0.042970793	0.05120911	3.54E-07	217598	0.000119153	25.9303398	FALSE	0.868199594
rs78746179	17	44204606	T	C	-0.221089057	0.042307383	0.07136733	1.73E-07	217598	0.000125485	27.308521	TRUE	0.551591959

SUI: stress urinary incontinence; SNP: single nucleotide polymorphism.

Table S13. Causality and sensitivity analysis of SUI and HDL-C in reverse two-sample MR analysis.

Exposure	Outcome	SNP	Methods	β	Standard Error	*P* -value	OR
SUI	HDL-C	7	MR Egger	-0.072843977	0.183987042	0.708494719	0.929745881
			Weighted median	0.000534605	0.074930292	0.994307385	1.000534748
			IVW	0.015253208	0.060401196	0.800630222	1.015370132
			Simple mode	0.014108397	0.10623186	0.898687637	1.01420839
			Weighted mode	0.008616366	0.100860204	0.93469984	1.008653594
			Cochran's Q statistic (heterogeneity P)		MR Egger intercept(pleiotropy *P*)		MR PRESSO *P*
			0.988884214		0.633770937		0.986

HDL-C: high density lipoprotein cholesterol; SUI: stress urinary incontinence; MR: mendelian randomization; IVW: inverse variance weighting; SNP: single nucleotide polymorphism.
